# Ant Mortality with Food Competition in Forests along a Temperature Gradient

**DOI:** 10.3390/insects13040392

**Published:** 2022-04-15

**Authors:** Tae-Sung Kwon, Dae-Seong Lee, Young-Seuk Park

**Affiliations:** 1 Alpha Insect Diversity Lab, Nowon, Seoul 01746, Korea; insectcom@naver.com; 2 Department of Biology, Kyung Hee University, Dongdaemun, Seoul 02447, Korea; dleotjd520@naver.com

**Keywords:** interspecific competition, intraspecific competition, body size, temperature effect, ant community, species interaction

## Abstract

**Simple Summary:**

Ants are aggressive, and many ants die from inter- or intraspecific attacks while acquiring food. Temperature influences animal behavior, including aggression and competition, but the effect of temperature on ant mortality due to food competition in the field remains unclear. We aimed to elucidate the relationship between temperature and mortality due to food competition in ant communities in forests. A field experiment was conducted using four bait types at six different oak forest sites with different mean annual temperatures along a temperature gradient. The results showed that the mortality rate due to food competition displayed a hump-shaped trend with temperature distribution being higher with intermediate temperatures and a linear trend increasing or decreasing with temperature along the temperature gradient. The mortality rate due to interspecific competition was higher than that due to intraspecific competition. The results indicated that mortality due to inter- or intraspecific competition for food was associated with temperature, density of other species, and species characteristics such as body size, dominance, feeding strategy, and aggressiveness.

**Abstract:**

The authors elucidated the relationship between temperature and mortality due to food competition in ant communities in forests. A field experiment was conducted using four bait types at six different oak forest sites with different mean annual temperatures in South Korea. The mortality rate due to food competition showed a hump-shaped trend, with temperature distribution being higher at study sites with intermediate temperatures and a linear trend increasing or decreasing with temperature along the temperature gradient. In most species, the mortality rate due to interspecific competition was higher than that due to intraspecific competition, but the dominant species, which were less affected by other species, had a higher mortality rate due to intraspecific competition. In subordinate species that are highly affected by other species, the mortality rate due to intraspecific competition increased as the mortality rate due to interspecific competition decreased. The results indicated that mortality due to inter- or intraspecific competition for food was associated by temperature, density of other species, and species characteristics (body size, dominance, feeding strategy, and aggressiveness). Given the relationship between temperature and mortality due to food competition, the authors expect that changes in competition due to climate warming will affect the fitness of ant species.

## 1. Introduction

Competition for food has often been observed in ants [1], and many worker ants are killed in competition for food resources [2,3]. Mortality due to food competition is an important threat to the survival of early ant colonies [4]. Competition is affected by various factors, including natural or anthropogenic disturbance and temperature [5,6]. Among them, temperature is expected to significantly affect competition by affecting the foraging and metabolic rate of species [5], and the intensity of competition varies by species. Ant activity levels and trophic preferences shift with temperature changes [7]. Seifert [8] was the first who showed that competitive exclusion in multi-species communities in nature exists and that interspecific competition increases with relatedness. Frizzi, et al. [9] revealed the role of environmental temperature in affecting the survival ability of an invasive garden ant, *Lasius neglectus*, in competition with other species. Cerdá, et al. [10] studied the role of competition with dominant species and temperature in the foraging of subordinate ant species and suggested that the foraging of subordinate species was influenced more by temperature than by competition with dominant species. Kwon [11] revealed that competition between ants for food varied with temperature in South Korea. Food competition was greatest at intermediate temperatures, decreasing at both low and high temperatures. The reason for this was that the species involved in intense competition for food were usually found at intermediate temperatures, and the prevalence of such species decreased when the temperature was lowered or higher. Therefore, mortality due to food competition is likely to be affected by temperature. However, few studies have examined the effect of temperature on mortality due to competition for food in ants in the field. Therefore, we aimed to test the hypothesis that mortality due to food competition would increase at intermediate temperatures in forest ant species. 

The co-occurrent ants, even closely related species, show different food preferences [12,13], and body size, aggression, recruitment behavior, and activity timing vary depending on the species [14,15]. Owing to differences in behavioral characteristics and body size between ant species, different ant colonies can consist of aggressive dominant species or submissive subordinate species [8,16]. A competitive hierarchy is created among ants gathered at large-sized food [17,18]. In competition for food, subordinate species can have a higher mortality rate than dominant species. In addition, it is expected that large-bodied individuals can kill small-bodied individuals more than vice versa. Food competition between ants is fierce not only between different species but also within the same species [19,20]. The intensity of inter- and intraspecific competition for food can vary between species. In particular, in the case of subordinate species, if the mortality rate due to interspecific competition decreases, then the mortality rate due to intraspecific competition is expected to increase. However, in dominant species that are less affected by other species, a significant relationship between interspecific competition mortality and intraspecific competition mortality would not be expected. 

To test these expectations, we investigated the mortality rate due to food competition among ants in six regions under different temperature conditions from the southernmost area to the northernmost area of South Korea.

## 2. Materials and Methods

### 2.1. Study Sites

**Figure 1:**
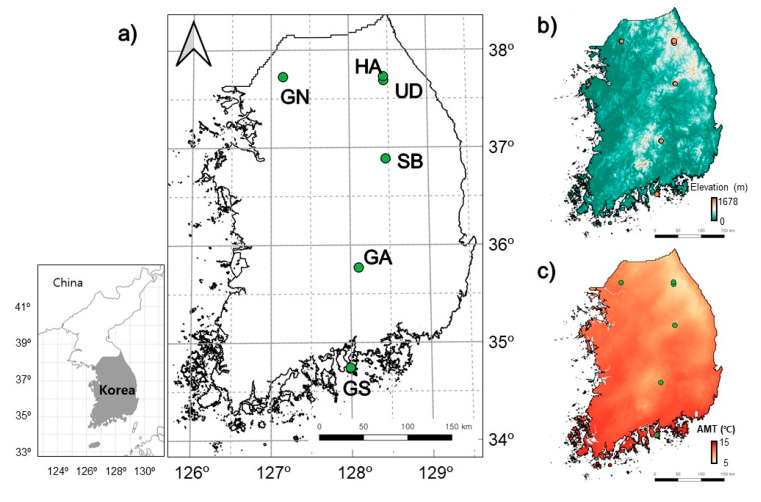
Study sites (**a**) and environmental conditions: elevation (**b**) and annual mean temperature (ATM) (**c**). UD: Unduryong, HA: Haanrim, GN: Gwangneung, SB: Sobaeksan, GA: Gayasan, and GS: Geumsan.

This study was carried out at six different oak forest sites (Unduryong: UD, Haanrim: HA, Gwangneung: GN, Sobaeksan: SB, Gayasan: GA, and Geumsan: GS) in South Korea (Figure 1), with mean annual temperatures (MATs) ranging from 7.4 °C in the northernmost area to 12 °C in the southernmost area [11]. MAT was measured with a data logger at 10 cm depth at each study site. The average distance between the survey sites was 181.1 ± 101.3 km. The field temperature was measured every 30 min during the study period (19 November 2013 to 18 November 2014) with a temperature data logger (Hobo VTBI/Hobo Tidbit v2, Onset Computer Cooperation, Bourne, MA, USA) placed at 10 cm depth in the soil at each study site. All the study sites were in deciduous forests (crown coverage 60–95%) in which understory vegetation was sparse to moderate (coverage approximately 5–50%), and litter covered the ground. The forests had secondary growth (30–40 years old) at all sites except for Gwangneung (GN) (>100 years old). The dominant tree species was *Quercus mongolica* Fisch. ex Ledeb. 1850 at all sites except for Geumsan (GS), where *Quercus serrata* Thunb. ex Murray, 1784 was the dominant tree species. The details of the study sites were presented by Kwon [11]. 

### 2.2. Field Experiment

Ground-foraging ants were used to investigate the mortality rate due to food competition among ants under different temperature conditions. They are usually omnivorous and are the most diverse ant species in South Korean forests [21]. A plot (15 m × 20 m) of 5 × 4 grid cells at 5-m intervals was created at each site using 20 small flags. The mortality of ants during food competition was studied using four different baits: canned silkworm pupae (*Bombyx mori* (Linnaeus, 1758)) (Dongwon Co., Changwon, Korea), cat food (Diamond Pet Food, Meta, USA), spongy cake (castella) (Shany Co., Seongnam, Korea), and honey (Dong Suh Foods, Jincheon, Korea). The spongy cake contained eggs and sugar; hence, its protein content was higher than that of general bread. Protein content was the highest in cat food (32%), and carbohydrate content was the highest in the spongy cake (12%). Three silkworm pupae (approximately 0.85 g) and similar volumes of the other baits were placed on each bait card for the experiment. 

The experiment with baits was performed with eight replicates from 10:00 to 16:00 on non-rainy days in the summer of 2013 and 2014. Twenty bait cards (four bait types and five replicates) in each plot were spaced 5 m apart in a fixed order (pupae, cat food, honey, and spongy cake from the first to fourth experiments, and honey, spongy cake, cat food, and pupae from the sixth to eighth experiments). Therefore, each type of bait was placed on five cards in a plot. The card was made of thick recycled paper (10 cm × 14.5 cm). 

After the bait cards were set, the number of ants visiting the cards was recorded at 10, 30, 50, 70, and 90 min. Ants visiting the cards were identified to the species level according to the South Korean ant identification key from Kwon, Kim, Lee, Jung and Sung [21], except for *Crematogaster* (*teranishi* Santschi, 1930 and *vagula* Wheeler, 1928) and *Myrmica* (*kotokui* Forel, 1911 and *kurokii* Forel, 1907), which were recorded as *Crematogaster* and *Myrmica* groups because of identification difficulties in the field. The number of ants that were killed or carried away by other ants was also recorded. The body weight of the ant species commonly observed at the bait cards was measured using an analytical balance (Mettler Toledo, XS205, Columbus, USA) after the ant bodies were dried at 55 °C for 72 h. During the baiting experiments, atmospheric temperature was measured using a mercury thermometer at the study sites. Data used in the study were provided in the Supplementary Materials Table S1.

### 2.3. Data Analyses

The authors tested the effect of MAT and atmospheric temperature (AT) on the mortality of ants due to food competition using a Poisson regression model [22]. Three abundant species (*Nylanderia flavipes* (Smith, 1874), *Aphaenogaster japonica* Forel, 1911, and *Pheidole fervida* Smith, 1874) that occurred at most study sites were mainly analyzed. The mortality of ants associated with four different baits was pooled at each study site to focus on the effects of temperature. The difference in mortality from inter- and intraspecific competition was evaluated, and total mortality from both inter- and intraspecific competitions was also analyzed. *N. flavipes* were subordinate during food competition, whereas the other two species were dominant [11]. 

Two temperature variables, AT and MAT, were expected to determine ant mortality due to food competition. Temperature influences the number of ants recruited and the intensity of competition among ants [4,11]. The mortality of each species was influenced by their abundance. Therefore, the mortality rate (d = [number of dead ants/number of ants] × 100) was calculated to remove this effect. In the Poisson regression models, the mortality rate of each species was used as a dependent variable, whereas AT, MAT, and MAT^2^ were used as independent variables. The minimum adequate model consisting only of variables with significance (*p* < 0.05) was compared with the full model using all three variables, AT, MAT, and MAT^2^. The minimum adequate models were created by sequentially removing the least significant variable from the full model [22]. The quasi-Poisson distribution was used when the residual deviance was more than twice the degree of freedom as a result of using the Poisson distribution. The effects of both ant body size and dominance on mortality were evaluated using the χ^2^ test. Statistical analyses were conducted using a package stat in R [23]. To test the hypothesis that the hump-shaped trend, with the highest mortality rate at the intermediate temperature, would occur, MAT was used as an explanatory variable.

## 3. Results

In bait experiments, 16 species with 134,030 ant individual observations were recorded (Table 1). *Myrmica* spp. and *Camponotus atrox* Emery, 1925 were mainly observed at low-temperature sites, whereas *A. japonica*, *Lasius japonicus* Santschi, 1941, and *P. fervida* were observed at most sites, showing a hump-shaped distribution with a larger number of individuals at intermediate temperatures. The most abundant species was *N. flavipes*, whose occurrence increased as a function of temperature. 

In the experiment, 173 individuals (0.13% of the total number of visitors) were killed in the context of intra- and inter-species competition for food. Small-bodied and abundant species, such as *N. flavipes* and *P. fervida*, had a high number of victims (i.e., killed ants) (Table 2 and Table 3). *N. flavipes*, the most abundant species, showed the highest number of victims with 76 individuals (44% of the total victims; 0.12% of its abundance), followed by *P. fervida*, the second most abundant species, with 61 individuals (35% of total victims; 0.16% of its abundance) (Table 2 and Table 4). Although the number of individuals killed in the context of interspecific competition was similar between the two species, the number of individuals killed in the context of intraspecific competition was higher in the subordinate species, *N. flavipes* (19 individuals), than in the dominant species, *P. fervida* (three individuals) (Table 2 and Table 4). Victims of *N. flavipes* due to intraspecific competition occurred mainly at the highest temperature site (89%), where the mortality due to interspecific competition was very low. Meanwhile, *Myrmica* spp. showed the highest victim rate at 0.53% (16 out of 2996 observed individuals). As the dead ants were transported to the nests of their killers, it is likely that they were used for food by inter- or intraspecific individuals (Table 5). 

*P. fervida* was the only species with soldier ants (5.4% of observed individuals). Among the dead ants, five soldiers were killed by *Formica japonica* and *A. japonica*, which were larger than *P. fervida* (Table 3). *A. japonica*, a dominant species with a relatively large body, was also frequently killed. The number of *A. japonica* individuals killed by intraspecific competition (12 individuals) was higher than that killed by interspecific competition (seven individuals) (Table 4). In the case of mortality due to interspecific competition, there were 131 cases in which large species killed small species, but only five cases in which smaller species killed larger species (χ^2^ = 116.7, *p* < 0.001; Table 2 and Table 3). There were also 131 cases in which dominant species killed subordinate species, but only four cases in which subordinate species killed dominant species (χ^2^ = 119.5, *p* < 0.001; Table 2 and Table 3). 

The mortality rate due to intraspecific competition was in the range of 0.01–0.1%, whereas that due to interspecific competition was in the range of 0.12–0.53%, indicating a higher mortality rate from interspecific competition (Table 4). *Myrmica* spp. had the highest mortality rate in general due to intra- and interspecific competition. In the subordinate species, *N. flavipes* and *Myrmica* spp., the interspecific competition mortality rate was 3 and 4.3 times higher than that of intraspecific competition, respectively. *P. fervida*, a small dominant species, showed a considerably higher interspecific mortality rate compared with that of intraspecific competition (19.3 times), whereas the intraspecific competitive mortality rate for *A. japonica*, a medium-sized dominant species, was twice that for interspecific competition. The mortality rate might be underestimated due to the repeated counts of same individuals.

**Figure 2:**
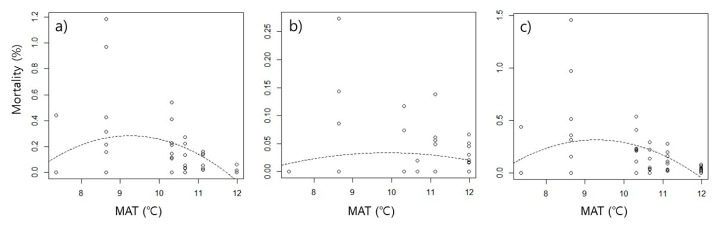
Mortality rate (%) of all ant species caused by food competition along the temperature gradient. (**a**) Interspecific competition, (**b**) intraspecific competition, and (**c**) total. The experiment was carried out with eight replicates at each temperature condition, and their mortality rates were presented in the scatter plot. The fitted lines are based on the quadratic regression models according to significant influences shown in Table 4. MAT: mean annual temperature.

**Figure 3:**
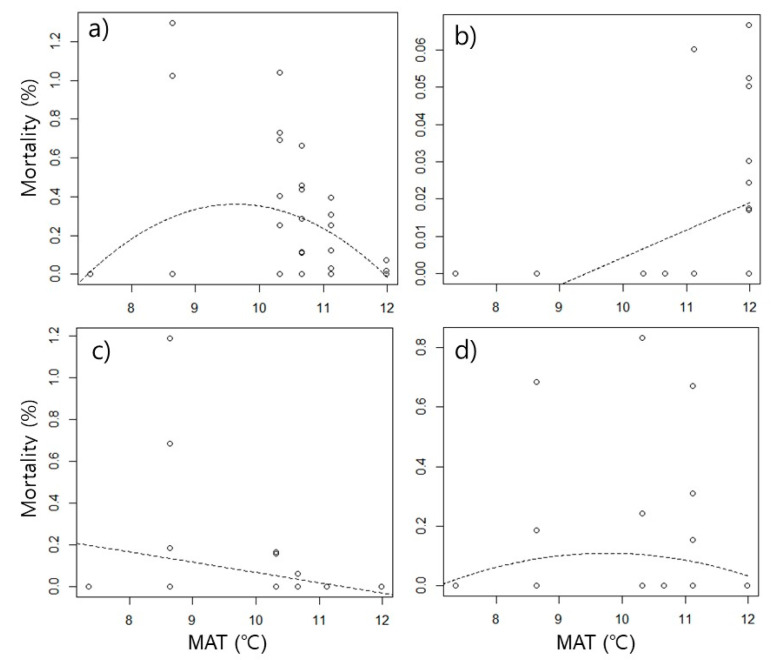
Mortality rate (%) of abundant ant species caused by food competition along temperature gradient. (**a**) Mortality of *Nylanderia flavipes* from interspecific competition, (**b**) mortality of *N. flavipes* from intraspecific competition, (**c**) mortality of *Aphaenogaster japonica* from interspecific competition, and (**d**) mortality of *A. japonica* from intraspecific competition. The experiment was performed with eight replicates at each temperature condition, and their mortality rates were presented in the scatter plot. The fitted lines are based on the linear or quadratic regression models according to significant influences shown in Table 6. MAT: mean annual temperature.

The mortality rate caused by food competition was higher at intermediate temperatures than at low and high temperatures, showing hump-shaped curves (Table 2, Figure 2). The peaks of mortality were observed around 9.3 ℃ in interspecific competition, 9.8 ℃ in intraspecific competition, and 9.3 ℃ in total competition. The significance of the hump-shaped trend was determined by the significant effect of MAT^2^ (*p* < 0.05) (Table 6). However, the trend differed depending on the species. In *N. flavipes*, interspecific competitive mortality showed a hump-shaped trend, but intraspecific competitive mortality showed a linear trend that increased as temperature increased (Figure 3a,b). Mortality due to interspecific competition in *P. fervida* displayed a hump-shaped trend (Table 6), but intraspecific competition was not analyzed because of the low frequency (n = 3). *A. japonica* showed a contrasting trend to *N. flavipes*. In interspecific competition, a linear trend that increased at low temperatures was observed (Figure 3c, Table 6), and in intraspecific competition, a hump-shaped trend was observed (Figure 3d, Table 6). The peaks of mortality were observed around 9.7 ℃ in interspecific competition of *N. flavipes*, and 9.8 ℃ in intraspecific competition of *A. japonica*.

## 4. Discussion

### 4.1. High Mortality at Intermediate Temperatures

The results show that ant mortality in the context of food competition follows a hump-shaped trend regarding habitat temperature, with the highest values at intermediate temperatures and lower values at lower or higher temperatures. The results support the hypothesis that the intensity of competition is higher at intermediate temperatures, following a hump-shaped trend [11]. The intensity of competition was calculated as the frequency of displacing the species occupying the bait cards. If the food competition was the most severe at the intermediate temperature, and thus the mortality rate is the highest, then a change in the mortality rate due to differences in temperature could be expected. If the temperature gradually increases, then the mortality due to food competition would increase in areas with lower temperatures than at intermediate temperatures but would decrease in areas with higher temperatures. 

The mortality caused by interspecific competition was considerably higher in small-bodied species attacked by large-bodied species, and in subordinate species attacked by dominant species. The mortality rate due to intra- and interspecific competition varied depending on the dominance and body size of the species. In most species, mortality due to interspecific competition was considerably higher than that due to intraspecific competition. Previous research has suggested that mortality caused by intraspecific competition was higher than that caused by interspecific competition because the same species use the same resources [24], differing from the results.

### 4.2. Different Mortality among Species

*N. flavipes* mainly competed with other species at intermediate temperatures, but primarily competed within the species at high temperatures. At the high-temperature site (11.98 °C site, GS) the abundance of the two dominant species *A. japonica* and *P. flavipes* was suppressed due to the high temperature, whereas the recruitment of *N. flavipes* to the baits greatly increased as a result of the lower competitive pressure accompanied by much higher intraspecific aggression (Table 1, Figure 3). Propensity for inter- or intraspecific aggression varies greatly among ant species with subordinate species tending to the latter [25]. This finding indicates that climate warming will lead to changes in competitive interactions between ants. *N*. *flavipes* workers in intermediate-temperature areas will have more opportunities to fight with intraspecific individuals in the future, whereas less aggressive (low competitive interactions) ant communities in low-temperature areas will struggle with the northward and upward shifts of *P. flavipes* and *A. japonica* [11]. 

*A. japonica* workers have relatively large bodies and forage either individually or in groups, whereas *P. fervida* workers have small bodies and mainly forage in groups. The mass-recruited small ant species tend to occupy large pieces of food more often than large ant species [18]. In this experiment, no clear distinctions in the willingness to fight were observed between majors and minors. However, majors attempted several times to displace *N. flavipes* workers from the honey bait, whereas the minors were feeding on the honey en masse. Interestingly, the avoidance behavior (retreating without the attacking opponent) that was most commonly found in *N. flavipes* (84% of 330 total contacts) was not observed in any *P. fervida* workers (0% of 120 contacts) [11]. Even the most dominant species, *C. atrox,* avoided 1 of 11 contacts. Of the species examined, *P. fervida* workers were the most likely to attack other individuals and did not flee from aggression, resulting in high interspecific mortality. Two small ant species were mostly killed by *A. japonica* (50% in *N. flavipes* and 84% in *P. fervida*). Seventeen percent of the killed *P. fervida* workers died in counter-attacks (i.e., first they attacked and then were killed by counter-attack) rather than in attacks (i.e., killed workers biting the legs of *A. japonica* or *L. japonicus*), indicating a trade-off between high food occupancy and high mortality. 

*N. flavipes* was the most subordinate species but also the most numerically dominant because it is the most common and abundant ant species in South Korea [11,21]. In the experiment, *N. flavipes* usually appeared at the bait cards first but then retreated as other species approached. In contrast to *P. fervida*, *N. flavipes* gave up occupied bait cards to avoid other ant species. Therefore, the ability to swiftly discover food and risk avoidance of *N. flavipes* likely explain parts of the ecological success. Ants modulate their behavior, specifically the tradeoff between food intake and mortality risk, to maximize fitness based on their phenotypic characteristics and social mechanisms [26,27]. *P. fervida* maximizes the former to optimize foraging, whereas *N. flavipes* minimizes mortality risk. The *N. flavipes* workers were explosively recruited in the absence of other species (Table 1). Therefore, the recruitment of *N. flavipes* seems to be highly dependent on the presence of other species. However, they did not avoid fighting with workers of the same species, despite their timidity toward other species. 

### 4.3. Effects of Climate Change on Species Distribution

The two *Myrmica* species (*kotokui* and *kurokii*) are currently numerically dominant at high altitudes in South Korea [28], but they are expected to decrease greatly in abundance as the climate warms [29]. As temperature increases, *A. japonica* is expected to gradually replace *Myrmica* spp. in high-altitude regions. *A. japonica* workers covered the honey bait with soil, small stones, and litter and carried these materials laden with honey. This behavior has also been observed in *Aphaenogaster rudis* by Enzmann (1947) in North America [30]. *Myrmica* and *Aphaenogaster* may increasingly compete with each other as the climate warms [29]. When *Myrmica* workers were encountered or attacked by the more dominant species (*A. japonica* and *P. fervida*), they remained motionless with folded legs and antennae. Attacks by opponents did not occur following the onset of this behavior (three cases with *A. japonica* and one case with *P. fervida*). This submissive behavior was termed the pupal position and has been reported in small Myrmicinae ant species [25]. However, these cold-adapted highland ants are more vulnerable to food competition given the highest mortality occurring during food gathering. *A. japonica* is a keystone species in the competitive interactions in South Korean ant assemblages with the suppression of *N. fervida* in high-temperature areas, the strong competition with *P. fervida* in intermediate-temperature areas, and the suppression of *Myrmica* species in low-temperature areas.

## 5. Conclusions

The mortality rate of ants due to food competition was between 0% and 0.53%. However, this was significantly underestimated due to the repeated counts of same individuals. Contrary to expectations, mortality due to interspecific competition was significantly higher than that due to intraspecific competition. However, the dominant species, which were less affected by other species, had a higher mortality rate due to intraspecific competition. In the case of the most subordinate species, *N. flavipes*, the mortality rate due to intraspecific competition increased sharply when mortality by other species decreased. The authors hypothesized that mortality due to food competition would decrease at intermediate and high temperatures but increase at low temperatures as climate warms, which was evidenced by the results. Given the effect of temperature on worker mortality due to food competition, it is expected that changes in competition due to climate change can affect the fitness of ant species in the future. The results of this study showed that the mortality of ants due to food competition is determined not only by physical environmental factors (temperature) but also by biological factors, such as the abundance and characteristics of species (body size, dominance, and aggressiveness).

## Figures and Tables

**Table 1:** Abundance (number of individuals) and occurrence frequency (percentage of occupied traps) of ants attracted by baits at six different temperature sites in South Korea. Temperature in parentheses is the annual mean temperature at each site. UD: Unduryong, HA: Haanrim, GN: Gwangneung, SB: Sobaeksan, GA: Gayasan, GS: Geumsan, Abund: abundance, and Occur: occurrence frequency.

Species	UD (7.35 °C)		HA (8.64 °C)		GN (10.32 °C)		SB (10.60 °C)		GA (11.12 °C)		GS (11.98 °C)	
	Abund	Occur	Abund	Occur	Abund	Occur	Abund	Occur	Abund	Occur	Abund	Occur
*Aphaenogaster japonica*	9	33.75	1692	40.63	5430	63.75	9153	79.38	6248	79.38	2	0.63
*Camponotus atrox*	242	59.38	246	48.75	26	8.75	0	0	0	0	0	0
*Camponotus nipponensis*	0	0	0	0	21	1.25	1	0.63	0	0	0	0
*Camponotus* sp.	0	0	0	0	0	0	0	0	1	0.63	0	0
*Crematogaster osakensis*	0	0	0	0	0	0	604	1.25	0	0	0	0
*Crematogaster vagula*	0	0	0	0	0	0	0	0	9	1.25	597	3.75
*Ectomomyrmex javanus*	0	0	0	0	0	0	0	0	7	3.75	117	21.25
*Formica japonica*	2	1.25	0	0	254	21.88	22	5	334	20.63	0	0
*Lasius japonicus*	28	6.88	1	0.63	1460	21.88	618	15.63	466	1.88	138	3.75
*Lasius spathepus*	214	0.63	0	0	0	0	0	0	0	0	0	0
*Myrmica* spp.	536	83.13	2460	78.75	0	0	0	0	0	0	0	0
*Nylanderia flavipes*	36	0.63	1074	25.00	2655	68.13	6927	95.63	12,882	94.38	42,289	100.00
*Pheidole fervida*	229	11.25	84	2.50	15,201	44.38	12,077	38.75	8484	23.13	946	5.63
*Themnothorax nassonovi*	199	35.00	0	0	2	0.63	0	0	0	0	0	0
*Vollenhovia emeryi*	0	0	0	0	0	0	1	0.63	0	0	1	0.63

**Table 2:** Number of ants killed from food competition at six different temperature sites. MAT: mean annual temperature.

MAT (°C)	Killer Species	Victim Species				
		*Lasius japonicus*	*Pheidole fervida*	*Myrmica* spp.	*Nylanderia flavipes*	*Aphaenogaster japonica*
7.35	*Camponotus atrox*			1		
8.64	*A. japonic* *a*		2	3	1	3
	*C. atrox*			9	6	4
	*Myrmica* spp.			3		
10.32	*A. japonica*		34		9	3
	*C. atrox*		6			1
	*F. japonica*		1			
	*P. fervida*				1	1
10.60	*A. japonica*	1	12		16	
	*F. japonica*				1	
	*L. japonicus*				2	
	*P. fervida*		1			1
11.12	*A. japonica*		3		12	6
	*F. japonica*				1	
	*N. flavipes*				2	
	*P. fervida*		2		5	
11.98	*L. japonicus*				2	
	*N. flavipes*				17	
	*Ectomomyrmex javanus*				1	
	SUM	1	61	16	76	19

**Table 3:** Weight (mean ± standard deviation) and dominance of ants. Dominance is the percentage of dominant behavior during interspecific encounters, and therefore, dominant species have a high score [11].

Species	Weight (mg)	N	Dominance
*Camponotus atrox*	7.942 (3.594)	22	87.2
*Ectomomyrmex javanus*	5.761 (0.694)	20	
*Formica japonica*	1.770 (0.642)	20	30.2
*Myrmica kotokui*	1.048 (0.124)	19	30.9
*Myrmica kurokii*	0.747 (0.172)	22	
*Aphaenogaster japonica*	0.679 (0.108)	19	84
*Pheidole fervida* (major)	0.375 (0.063)	30	79.7
*Pheidole fervida* (minor)	0.107 (0.018)	25	
*Lasius japonicus*	0.211 (0.075)	24	50.3
*Crematogaster vagula*	0.185 (0.045)	2	
*Crematogaster matsumurai*	0.165 (0.035)	27	
*Vollenhovia emeryi*	0.108 (0.018)	5	
*Themnothorax nassonovi*	0.085 (0.026)	40	
*Nylanderia flavipes*	0.068 (0.017)	26	3.6

**Table 4:** Mortality (number of individuals (N) and percentage (%)) of ants from food competition.

Species	Intraspecific Competition	Interspecific Competition	Total
*Aphaenogaster japonica*	12 (0.05)	7 (0.03)	19 (0.08)
*Lasius japonicus*		1 (0.04)	1 (0.04)
*Myrmica* spp.	3 (0.10)	13 (0.43)	16 (0.53)
*Nylanderia flavipes*	19 (0.03)	57 (0.09)	76 (0.12)
*Pheidole fervida*	3 (0.01)	58 (0.16)	61 (0.16)

**Table 5:** Number of killed ants carried by other ants.

Killer Species	Victim Species				
	*Lasius japonicus*	*Pheidole fervida*	*Myrmica* spp.	*Nylanderia flavipes*	*Aphaenogaster japonica*
*L. japonicus*				2	
*P. fervida*		2		4	
*Myrmica* spp.			2		
*N. flavipes*				11	
*A. japonica*	1		2	2	5

**Table 6:** Results of Poisson regression model on relationships between mortality from food competition and temperature. AT: atmospheric temperature measured 1 m above ground during survey; MAT: mean annual temperature measured by data logger at 10 cm depth.

Victim Species	Killer	Variable	Full Model		Minimum Adequate Model	
			Coef.	AIC	Coef.	AIC
*Nylanderia flavipes*	Other species	AT	0.13 *	126.4	0.13 *	126.4
		MAT	9.977 ***		9.977 ***	
		MAT^2^	−0.548 ***		−0.548 ***	
	Same species	AT	−0.04 ns	36.6		33.5
		MAT	0.063 ns		2.213 *	
		MAT^2^	−0.274 ns			
	Total	AT	0.067 ns	136.1		137.2
		MAT	4.44 *		4.879 *	
		MAT^2^	−0.257 **		−0.275 **	
*Pheidole fervida*	Other species	AT	0.2 ***	87	0.2 ***	87
		MAT	14.758 **		14.758 **	
		MAT^2^	−0.838 ***		−0.838 ***	
	Same species	AT	0.114 ns	24		
		MAT	0.001 ns			
		MAT^2^	−0.633 ns			
	Total	AT	0.195 ***	24	0.195 ***	24
		MAT	11.463 **		11.463 **	
		MAT^2^	−0.654 **		−0.654 **	
*Aphaenogaster japonica*	Other species	AT	0.247 ns	35.4		34.5
		MAT	21.265 ns		−0.075 ***	
		MAT^2^	−1.21 ns			
	Same species	AT	0.267 *	34.5	0.267 *	34.5
		MAT	−13.532 *		−13.532 *	
		MAT^2^	0.65 *		0.65 *	
	Total	AT	0.249 *	78	0.245 *	79.5
		MAT	−7.922 ns		−1.343 ***	
		MAT^2^	0.342 ns			
Total	Other species	AT	0.182 ***	NE	0.182 ***	NE
		MAT	7.732 ***		7.732 ***	
		MAT^2^	−0.444 ***		−0.444 ***	
	Same species	AT	0.007 ns	110.4		108.4
		MAT	−4.457 *		−4.466 *	
		MAT^2^	0.217 *		0.218 *	
	Total	AT	0.128 **	NE	0.128 **	NE
		MAT	3.337 *		3.337 *	
		MAT^2^	−0.205 *		−0.205 *	

ns: *p* > 0.05, *: *p* < 0.05, **: *p* < 0.01, ***: *p* < 0.001, NE: not estimated.

## Data Availability

The original contributions presented in the study are included in the article/supplementary material, further inquiries can be directed to the corresponding author’s.

## Supplementary Materials

The following supporting information can be downloaded at: https://www.mdpi.com/article/10.3390/insects13040392/s1, Table S1: Data used in the study.
