# Supplementary material for: Ant Mortality with Food Competition in Forests along a Temperature Gradient
Source: Insects. 2022 Apr 15;13(4):392. doi: 10.3390/insects13040392 (PMC9032073; doi:10.3390/insects13040392)
Supplement: Supplementary file 1 [file insects-13-00392-s001.zip › insects-1677070-supplementary.pdf]

Supplementary Material

Ant mortality with food competition in forests along a temperature gradient

Tae-Sung Kwon[1], Dae-Seong Lee[2], Young-Seuk Park[2*]

[1]Alpha Insect Diversity Lab, Nowon, Seoul 01746, Republic of Korea

[2]Department of Biology, Kyung Hee University, Dongdaemun, Seoul 02447, Republic of Korea

*** Correspondence:** parkys@khu.ac.kr

Table S1. Data used in the study

Site	at	mat	do	ds	d	ab	pdo	pds	pd	p	ndo	nds	nd	n	ado	ads	ad	a
UD	19.5	7.35	0	0	0	222	0	0	0	11	0	0	0	0	0	0	0	0
UD	23.6	7.35	0	0	0	337	0	0	0	50	0	0	0	36	0	0	0	0
UD	23.2	7.35	0	0	0	257	0	0	0	45	0	0	0	0	0	0	0	0
UD	21.8	7.35	1	0	1	227	0	0	0	51	0	0	0	0	0	0	0	0
UD	19	7.35	0	0	0	310	0	0	0	3	0	0	0	0	0	0	0	0
UD	20.3	7.35	0	0	0	70	0	0	0	48	0	0	0	0	0	0	0	5
UD	18.3	7.35	0	0	0	49	0	0	0	14	0	0	0	0	0	0	0	3
UD	18.5	7.35	0	0	0	23	0	0	0	7	0	0	0	0	0	0	0	1
HA	20	8.64	0	0	0	511	0	0	0	0	0	0	0	25	0	0	0	131
HA	20.3	8.64	3	2	5	1392	2	0	2	78	0	0	0	298	0	0	0	71
HA	20.8	8.64	13	3	16	1096	0	0	0	1	4	0	4	391	2	2	4	292
HA	19.8	8.64	5	1	6	1169	0	0	0	0	3	0	3	231	1	1	2	540
HA	17.5	8.64	2	0	2	206	0	0	0	0	0	0	0	31	1	0	1	84
HA	18.9	8.64	0	0	0	236	0	0	0	0	0	0	0	54	0	0	0	97
HA	18	8.64	1	0	1	317	0	0	0	5	0	0	0	3	0	0	0	140
HA	17.8	8.64	1	0	1	630	0	0	0	0	0	0	0	41	0	0	0	337
GN	24	10.32	1	1	2	854	0	0	0	135	1	0	1	96	0	1	1	411
GN	23	10.32	4	0	4	1890	4	0	4	1079	0	0	0	245	0	0	0	541
GN	25	10.32	17	0	17	3152	16	0	16	2284	1	0	1	396	0	0	0	456
GN	27.5	10.32	13	0	13	3158	10	0	10	2194	2	0	2	289	1	0	1	629
GN	19	10.32	0	0	0	4221	0	0	0	1137	0	0	0	91	0	0	0	1655
GN	25	10.32	8	0	8	3507	5	0	5	2165	3	0	3	410	0	0	0	889
GN	21.5	10.32	6	0	6	5541	2	0	2	4172	3	0	3	746	1	0	1	609
GN	23	10.32	4	2	6	2726	4	0	4	2035	0	0	0	382	0	2	2	240

SB	24.5	10.66	1	0	1	1831	0	0	0	584	1	0	1	875	0	0	0	279
SB	24.6	10.66	1	0	1	3151	0	0	0	1024	1	0	1	914	0	0	0	969
SB	27	10.66	14	1	15	5125	4	1	5	2489	9	0	9	1358	0	0	0	1089
SB	24.3	10.66	10	0	10	4490	4	0	4	1327	5	0	5	1149	1	0	1	1605
SB	17.5	10.66	2	0	2	5424	1	0	1	2502	1	0	1	218	0	0	0	2612
SB	25.5	10.66	0	0	0	2668	0	0	0	919	0	0	0	790	0	0	0	900
SB	21.3	10.66	0	0	0	3054	0	0	0	1163	0	0	0	922	0	0	0	814
SB	19.6	10.66	5	0	5	3660	3	0	3	2069	2	0	2	701	0	0	0	885
GA	25	11.12	3	3	6	2167	0	0	0	657	3	0	3	981	0	3	3	447
GA	24.96	11.12	2	2	4	3590	0	0	0	1256	2	1	3	1660	0	1	1	651
GA	26.9	11.12	6	2	8	4065	1	0	1	2040	5	0	5	1259	0	2	2	644
GA	23.3	11.12	1	2	3	3287	1	2	3	976	0	0	0	1199	0	0	0	1045
GA	18	11.12	1	0	1	3102	0	0	0	1183	1	0	1	393	0	0	0	1193
GA	27	11.12	1	0	1	3260	1	0	1	1087	0	0	0	1486	0	0	0	498
GA	21.8	11.12	7	0	7	4370	0	0	0	569	7	0	7	2751	0	0	0	1049
GA	19	11.12	1	0	1	4590	0	0	0	666	1	0	1	3158	0	0	0	766
GS	23.5	11.98	0	1	1	3321	0	0	0	0	0	1	1	3303	0	0	0	2
GS	28.3	11.98	3	1	4	4798	0	0	0	477	3	1	4	4093	0	0	0	0
GS	30.5	11.98	0	2	2	4399	0	0	0	180	0	2	2	3809	0	0	0	0
GS	25	11.98	0	1	1	6106	0	0	0	212	0	1	1	5838	0	0	0	0
GS	20	11.98	0	0	0	2213	0	0	0	76	0	0	0	2106	0	0	0	0
GS	28.8	11.98	1	1	2	5806	0	0	0	0	1	1	2	5706	0	0	0	0
GS	22	11.98	0	5	5	7496	0	0	0	1	0	5	5	7487	0	0	0	0
GS	19.1	11.98	0	5	5	9951	0	0	0	0	0	5	5	9947	0	0	0	0

Variable	Content
Site	Sites
at	atmosphere temperature
mat	mean annual temperature
do	number of ants killed by other species
ds	number of ants killed by same species
d	number of ants killed
ab	number of ants gathered at baits
pdo	number of *Pheidole fervida* killed by other species
pds	number of *Pheidole fervida* killed by same species
pd	number of *Pheidole fervida* killed
p	number of *Pheidole fervida* gathered at baits
ndo	number of *Nylanderia flavipes* killed by other species
nds	number of *Nylanderia flavipes* killed by same species
nd	number of *Nylanderia flavipes* killed
n	number of *Nylanderia flavipes* gathered at baits
ado	number of *Aphaenogaster japonica* killed by other species
ads	number of *Aphaenogaster japonica* killed by same species
ad	number of *Aphaenogaster japonica* killed
a	number of *Aphaenogaster japonica* gathered at baits
